# Pulmonary hypertension with massive megalosplenia: A case report

**DOI:** 10.1097/MD.0000000000014594

**Published:** 2019-03-22

**Authors:** Tieci Yi, Wei Ma, Jianxing Qiu, Wenhui Ding

**Affiliations:** a Department of Cardiology; b Medical Imaging Department, Peking University First Hospital, Beijing, China.

**Keywords:** megalosplenia, myelofibrosis, myeloproliferative neoplasm, portal vein hypertension, pulmonary hypertension

## Abstract

**Rationale:**

Pulmonary hypertension (PH) is a complicated disease which has complex causes and poor outcome. Many factors are involved in the increase of pulmonary artery pressure. It is often difficult to identify the specific cause of a particular patient. However, identifying the etiology is of great importance for specifying treatment strategies and improving the prognosis of patients.

**Patient concerns:**

A 58-year-old male was admitted because of fatigue, breath shortness for 6 months, which got worse in the last 3 months. The ultrasound cardiogram (UCG) indicated a remarkably elevated pulmonary artery systolic pressure (PASP = 82 mm Hg). He had hypertension for 15 years. Besides, his spleen was found to be enlarged since 15 years ago. Bone marrow biopsy of the patient revealed myeloproliferative neoplasm (MPN) with severe myelofibrosis (MF).

**Diagnosis:**

Myeloproliferative neoplasm (MPN) with severe myelofibrosis (MF) which in turn caused PH and portal vein hypertension (PVH).

**Interventions:**

We treated the patient with diuretics and fosinopril, and also steroids and thalidomide for his MPN/MF.

**Outcomes:**

Two weeks later, the pulmonary artery pressure (PAP) was remarkably decreased (PASP = 53.1 mm Hg by UCG, mean PAP = 21 mm Hg by right cardiac catheterization). Within 2 years’ follow-up, his circulatory state and hematological state remained stable.

**Lessons:**

It is often difficult to define the cause of PH, but it is important for making the appropriate treatment at the same time.

## 1 Introduction

Pulmonary hypertension (PH) has had a significant increase in attention over the recent years. This has been driven simultaneously by the advent of new treatments for this condition and new insights into the genetic and pathological drivers of the disease. However, despite current therapeutics, the 3-year survival rate is about 50% to 70%, illustrates the ongoing challenges of managing this disease.^[1–5]^ The poor prognosis of PH is partially due to its diverse etiology and complex pathophysiological mechanisms. There are hundreds of causes for PH,^[6,7]^ and the therapies for PH caused by different causes are significantly different. Therefore, determining the cause for PH of a certain patient is critical to make the most optimal treatment plan for him.^[8,9]^ However, to identify the causes of PH is often a challenging task in clinical practice. Careful analysis of the patient's condition is crucial to make clear the real etiology of the patient.

## 2 Case presentation

**Figure 1:**
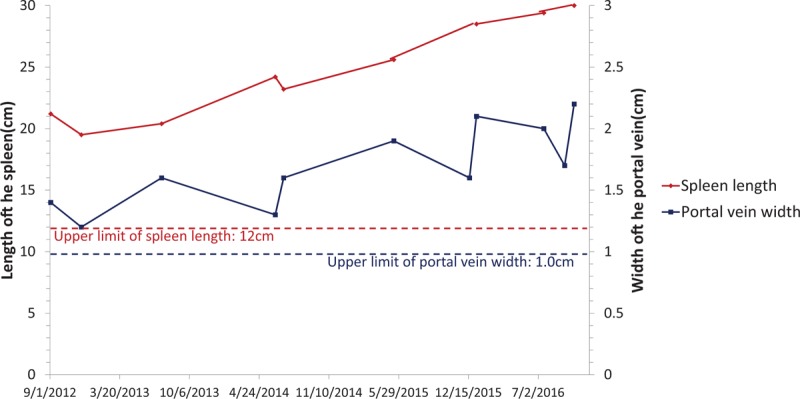
The changes of portal vein and spleen of the patient.

A 58-year-old male was admitted to the hospital because of fatigue, breath shortness for 6 months, and got worse in the last 3 months. He had taken an ultrasound cardiogram (UCG) in another hospital 1 week before admission which suggested a mildly enlarged left heart (left ventricular end-diastolic diameter [LVEDD] = 55 mm) with an otherwise normal left ventricular ejection fraction (54%) and a significantly enlarged right heart (right ventricular end-diastolic diameter [RVEDD] = 50 mm). The UCG also indicated a remarkably increased pulmonary artery systolic pressure (PASP = 82 mm Hg), accompanied by a widened inferior vena cava (22 mm) with decreased compression during inspiratory (<50%). After taking furosemide 20 mg Qd, spironolactone 20 mg Qd, and captopril 6.25 mg Bid for 1 week, the patient did not feel better. Previously, he had hypertension for 15 years. His spleen was found to be enlarged 15 years ago, and was referred to as “massive splenomegaly” since 6 years ago. Four years ago, his portal vein was found to be widened during abdominal ultrasound examination. However, examinations such as abdominal ultrasound and CT had not identified the cause for these abnormalities. He was not a smoker, but he used to drink about 100 g alcohols per day for more than 30 years, which he just quitted 2 months ago. The changes of his portal vein, spleen vein, and spleen are shown in Figure 1.

**Figure 2:**
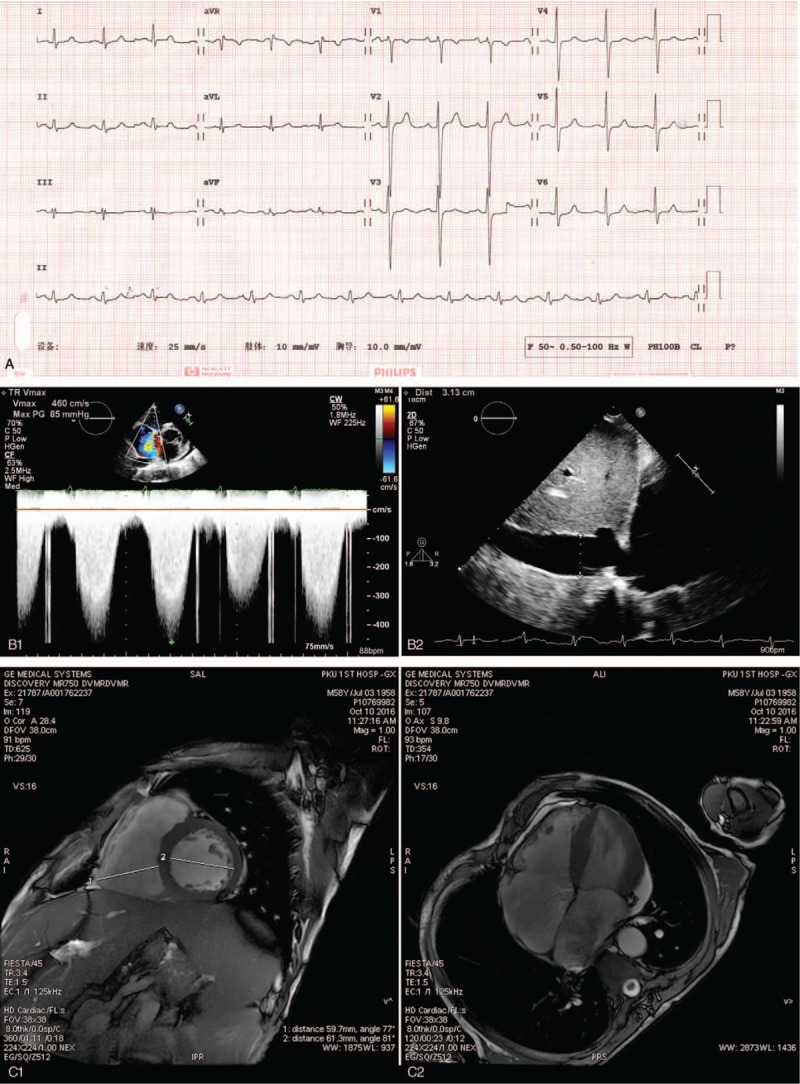
The results of ECG, UCG, and cardiac enhanced MR. A, It showed an otherwise normal ECG. B1, It showed the Doppler spectrum of the tricuspid valve regurgitation and the estimated tricuspid valve transvalvular pressure. B2, It showed the widened inferior vena cava, which suggested increased inferior vena cava and right atrial pressure. Combined B1 and B2, we can estimate the PASP. (C1) and (C2) were imagines of CEMR, and show the significantly enlarged heart.

On admission, we found his enlarged spleen had reached the right lower abdomen. His lower extremities had moderate edema on both sides. The ECG (Fig. 2A) and cardiac markers test of the patient were otherwise normal. In order to get further information, we had another UCG which also suggested heart enlargement (LVEDD 58 mm, RVEDD 49 mm). But, we also found the thickness of his ventricular wall was slightly increased (left ventricular free wall and interventricular septum: 12 mm; right ventricular free wall: 0.6 cm). An increased main pulmonary artery diameter (36 mm by UCG) and significantly increased pulmonary artery pressure (PASP>90 mm Hg by UCG, Fig. 2B1, B2) had also been proved. Furthermore, the UCG detected slightly decreased systolic function of the RV (tricuspid annular plane systolic excursion, TAPSE=1.1 cm). A cardiac enhanced MR (CEMR, Fig. 2C1, C2) found abnormal delayed enhancement only in the root of the anterior papillary muscle, while other myocardial muscles seem to be normal. What is more, the cardiac output (CO) measured by the CEMR reached as 9.0 L/min.

**Figure 3:**
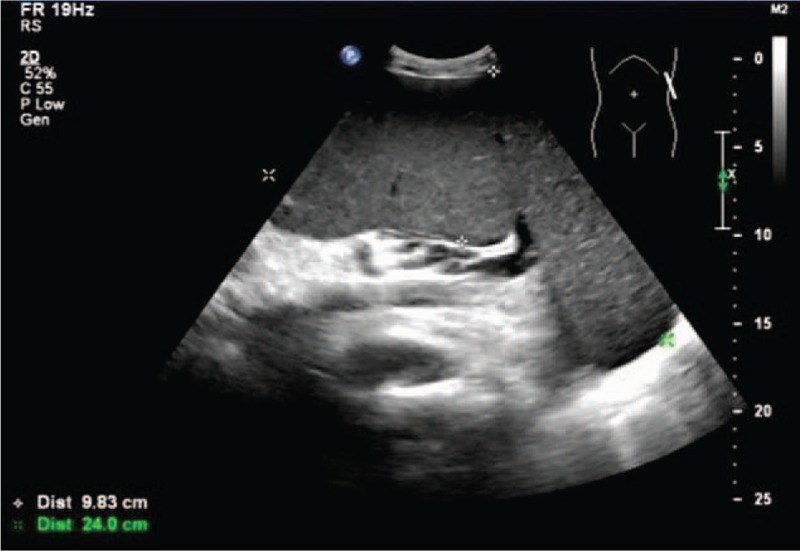
The B-mode ultrasonography of spleen.

As for other test results, the routine blood test showed a mild anemia (hemoglobin 105 g/L) and increased platelets (458×10^9^/L) and basophile (0.8 × 10^9^/L). His creatinine, transaminase, bilirubin and albumin levels were within normal limits, while LDH was increased (758IU/L). The abdominal ultrasound (Fig. 3) also found his spleen had entered the pelvic cavity (the intercostal thickness of spleen was 7.9 cm). An enlarged liver (oblique diameter of the right liver lobe 18.2 cm) and widened portal vein (1.8 cm) had also been found. Meanwhile, no signs of thrombosis could be found in the portal vein and inferior vena cava. Our imaging expert consulted the patient's previous abdominal enhanced CT and other images and came to similar conclusions.

**Figure 4:**
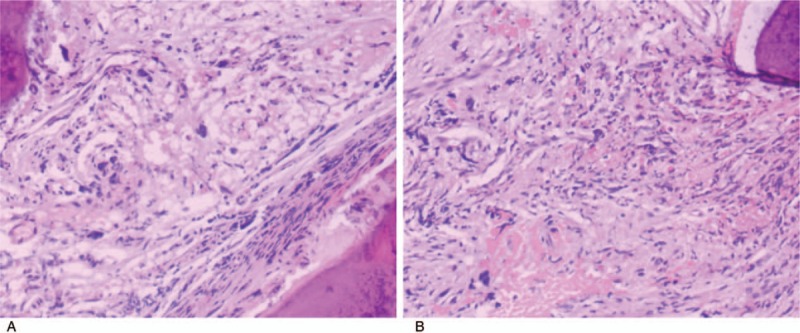
The result of his bone marrow biopsy. The bone marrow biopsy revealed significant hyperplasia of interstitial fibrous tissue and reticular fiber, and also hyperplasia of megakaryocyte, which distributed in a cluster. Naked megakaryocytes were visible. In conclusion, the patient had myeloproliferative neoplasms with severe myelofibrosis.

As his clinical manifestations and examination results indicated severe pulmonary hypertension and right heart failure, we tried to identify the cause of these abnormalities. We excluded structural left heart diseases, serious lung diseases, hypoxia, severe chronic pulmonary embolism and other pulmonary artery obstructions by UCG, CEMR and coronary CTA, pulmonary function test, arterial blood gas test and pulmonary ventilation and perfusion scan. However, the PVH and massive splenomegaly of the patient attracted our attention. As for the reason for his PVH, the analysis of hepatic venous pressure gradient (inferior vena cava pressure = 9mmHg, hepatic vein pressure = 9mmHg, wedged hepatic vein pressure = 15mmHg, hepatic vein pressure gradient = 6mmHg) suggested that it was a hepatic portal hypertension. The most common cause of hepatic portal hypertension is cirrhosis. Although the patient had a long history of heavy drinking (100 g alcohol per day for 30 years), his biochemical tests and abdominal imaging showed no evidence of cirrhosis. Non-cirrhotic hepatic portal hypertension always a headache problem in clinical practice. However, the high platelet and basophil cells, high LDH and the massive splenomegaly led us to consider about hematological problems. A further analysis of his peripheral blood smears found the percentage of the myelocyte and the metamyelocyte were 8% and 2%. The results of bone marrow biopsy (shown in Fig. 4) showed myeloproliferative neoplasm (MPN) with severe myelofibrosis (MF). The result of his gene analysis showed the expression of a JAK2 (Janus kinase 2) mutation, V617F, which also supported the diagnosis of MPN.

We treated the patient with furosemide, spironolactone and fosinopril in order to reduce the volume load of the patient and prevent cardiac remodeling. Steroids and thalidomide were given to the patient for his MPN and MF. Because of economic problem, the patient rejected to use targeting drugs for PH and MPN/MF.

2 weeks later, before his release from hospital, his symptom significantly released, and his pulmonary artery pressure was remarkably decreased (PASP=53.1 mm Hg by UCG; mean PAP=21 mm Hg, systolic PAP=34 mm Hg, diastolic PAP=15 mm Hg by right cardiac catheterization). During the 2-year follow-up, his symptom circulatory state and hematological state remained stable.

## 3 Discussion

This patient presented with severe edema and dyspnea which is considered to be associated with PH. PH is a complex clinical problem with various etiologies and unfavorable prognosis. Identifying the causes of PH is important for making the best therapy plan. However, it is difficult to determine the causes of PH sometimes. For this patient, we found that he had MPN and MF, which had caused his massive splenomegaly and PVH, and also probably was the cause of his PH, as no other reason of PH was found.

**Figure 5:**
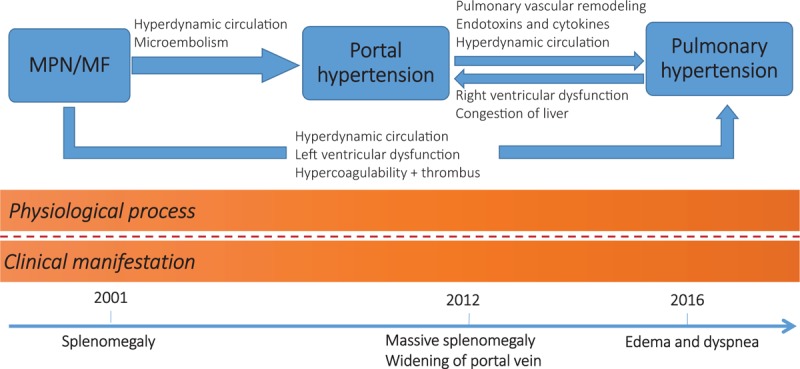
The flow chart of the progress and pathogenesis of the patient.

The relationship between MPN/MF and PH has long been discovered, but still not been fully understood. The prevalence of PH in the context of MPNs was still poorly defined. It ranged from 5% to about 50% among different studies, while in most studies it is about 40%.^[10–17]^ Precapillary PH in patients affected by MPN/MF may be caused by several factors: PVH, pulmonary veno-occlusive disease, tumor microembolism, pulmonary myeloid infiltration, enhanced angiogenesis, and a hypermetabolic state with high cardiac output and left ventricular dysfunction.^[16,18–20]^ PVH is a common complication and seen in up to 17% of patients of MPN with MF.^[16,21]^ It is reported that patients with PVH caused by MPN/MF have some unique characteristics compared with PVH caused by other reasons: normal liver function (normal albumin level), splenomegaly but no hypersplenism (normal white blood cell and platelet count), and immature blood cells can be seen in peripheral blood.^[22]^ All these features were present in this patient. Hyperdynamic circulation and high cardiac output were an important change of this patient. This change was also reported in other studies about MPN/MF.^[16,23]^ The causes of hyperdynamic circulation include excessive blood volume caused by extramedullary hematopoiesis, and also anemia. It explained the generally enlarged and slightly thickened left and right ventricular of this patient. More importantly, hyperdynamic circulation could be a cause of both PVH and PH and is commonly seen in portopulmonary hypertension.^[24]^ Overall, the progress and pathogenesis of the patient can be seen in Figure 5.

The treatment strategy for MPN/MF patients with PH remains controversial. For these patients, we should pay attention to their MPN/MF and PH at the same time. Conventional agents for MPN/MF, such as hydroxyurea and oral alkylating agents, may reduce splenomegaly in some patients, but its benefit is usually of short duration and tolerability is poor.^[18,19,25]^ Ruxolitinib is currently the only approved treatment for patients with MF that has been shown in pivotal randomized clinical trials to be highly effective in alleviating symptom burden and splenomegaly.^[26,27]^ However, it may have an adverse effect on pulmonary hypertension .^[28,29]^ The combination of thalidomide and prednisone has been evaluated in patients with MF and has shown remarkable efficiency.^[30]^ Splenectomy and transjugular intrahepatic shunt (TIPS) are treatments that can be considered for splenomegaly and portal hypertension caused by MF.^[31–33]^ However, both splenectomy^[34,35]^ and TIPS^[36,37]^ seems to have adverse effects on pulmonary circulation on long-term follow up, which limited their application in patients with elevated pulmonary pressure. Splenic irradiation is a palliative treatment for symptomatic splenomegaly due to myelofibrosis ^[25,38,39]^, which may also have a positive effect on pulmonary hypertension.^[40]^ Hematopoietic stem cell transplantation might also be a resolution of myelofibrosis-associated PAH.^[41]^

For this patient, we gave him diuretics to reduce his circulation volume, thalidomide for his MPN/MF. During the 2-year follow-up, his symptoms were stable, and the pulmonary artery systolic pressure measured by UCG remained at 40 to 50 mm Hg.

In general, it is a rare condition that PH coexisted with massive splenomegaly. In this case, after having MPN combined with severe MF without appropriate treatment for more than 10 years, the patient got massive splenomegaly. Under the influence of high volume, PVH and other possible factors, the patient developed PH. This case suggests that to find out the final cause of PH is often difficult, but important for providing the best therapy for patients.

## Acknowledgments

The authors also thank all who helped during the writing of this thesis.

## Author contributions

**Funding acquisition:** Tieci Yi.

**Investigation:** Tieci Yi, Wei Ma, Jianxing Qiu, Wenhui Ding.

**Resources:** Tieci Yi.

**Software:** Jianxing Qiu.

**Supervision:** Wei Ma, Wenhui Ding.

**Writing – original draft:** Tieci Yi.

**Writing – review & editing:** Tieci Yi.

Tieci Yi orcid: 0000-0001-9080-7745.
